# HIF-1α-PDK1 axis-induced active glycolysis plays an essential role in macrophage migratory capacity

**DOI:** 10.1038/ncomms11635

**Published:** 2016-05-18

**Authors:** Hiroaki Semba, Norihiko Takeda, Takayuki Isagawa, Yuki Sugiura, Kurara Honda, Masaki Wake, Hidenobu Miyazawa, Yoshifumi Yamaguchi, Masayuki Miura, Dana M. R. Jenkins, Hyunsung Choi, Jung-whan Kim, Masataka Asagiri, Andrew S. Cowburn, Hajime Abe, Katsura Soma, Katsuhiro Koyama, Manami Katoh, Keimon Sayama, Nobuhito Goda, Randall S. Johnson, Ichiro Manabe, Ryozo Nagai, Issei Komuro

**Affiliations:** 1 Department of Cardiovascular Medicine, Graduate School of Medicine, The University of Tokyo, Tokyo 113-8655, Japan; 2 Department of Cardiovascular Medicine, The Cardiovascular Institute, Tokyo 106-0031, Japan; 3 PRESTO, JST, Saitama 332-0012, Japan; 4 Department of Biochemistry, Keio University School of Medicine, Tokyo 160-8582, Japan; 5 Department of Genetics, Graduate School of Pharmaceutical Sciences, The University of Tokyo, Tokyo 113-8655, Japan; 6 Core Research for Evolutional Science and Technology, Japan Agency for Medical Research and Development, Tokyo 332-0012, Japan; 7 Department of Biological Sciences, The University of Texas at Dallas, Richardson, Texas 75080, USA; 8 Innovation Center for Immunoregulation and Therapeutics, Graduate School of Medicine, Kyoto University, Kyoto 606-8501, Japan; 9 Department of Physiology, Development and Neuroscience, University of Cambridge, Cambridge CB2 1SZ, UK; 10 Department of Life Science and Medical BioScience, School of Advanced Science and Engineering, Waseda University, Tokyo 162-8480, Japan; 11 Jichi Medical University, Tochigi 329-0498, Japan

## Abstract

In severely hypoxic condition, HIF-1α-mediated induction of *Pdk1* was found to regulate glucose oxidation by preventing the entry of pyruvate into the tricarboxylic cycle. Monocyte-derived macrophages, however, encounter a gradual decrease in oxygen availability during its migration process in inflammatory areas. Here we show that HIF-1α-PDK1-mediated metabolic changes occur in mild hypoxia, where mitochondrial cytochrome *c* oxidase activity is unimpaired, suggesting a mode of glycolytic reprogramming. In primary macrophages, PKM2, a glycolytic enzyme responsible for glycolytic ATP synthesis localizes in filopodia and lammelipodia, where ATP is rapidly consumed during actin remodelling processes. Remarkably, inhibition of glycolytic reprogramming with dichloroacetate significantly impairs macrophage migration *in vitro* and *in vivo*. Furthermore, inhibition of the macrophage HIF-1α-PDK1 axis suppresses systemic inflammation, suggesting a potential therapeutic approach for regulating inflammatory processes. Our findings thus demonstrate that adaptive responses in glucose metabolism contribute to macrophage migratory activity.

## Abstract (web summary)

Migration to the inflamed tissue demands energy production in an increasingly hypoxic environment. Here the authors show that during migration, HIF1α-induced PDK1 uniquely adapts macrophage metabolism to mild hypoxia by promoting glycolysis while preserving cytochrome c oxidase activity.

## Graphical abstract

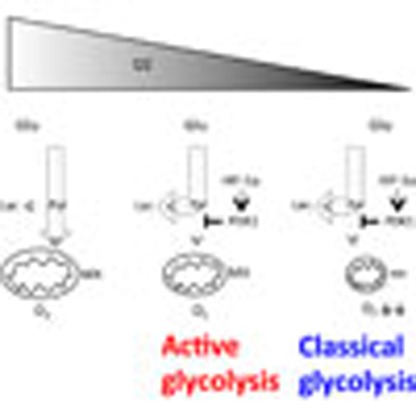

A cancer cell exhibits glycolysis even in the presence of oxygen, a phenomenon called the Warburg effect. In contrast, a non-cancer cell changes its cellular metabolism from glucose oxidation to glycolysis depending on the environmental oxygen concentration. Acting as a key enzyme in glucose oxidation, mitochondrial cytochrome *c* oxidase is the last oxidase of the mitochondrial electron transport chain and converts molecular oxygen to water. Mitochondrial cytochrome *c* oxidase consumes the majority of intracellular molecular oxygen. Furthermore, *in vitro* experiments revealed that the activity of cytochrome *c* oxidase activity decreases when the oxygen concentration drops below 1.0 μM (ref. 1). Therefore, the decrease in mitochondrial oxygen availability may account for the suppression of cytochrome *c* oxidase activity in severely hypoxic conditions.

In this study, we evaluate the glucose metabolism of primary macrophages, and demonstrate that HIF-1α-induced pyruvate dehydrogenase kinase, isozyme 1 (PDK1) promotes glycolysis in mild hypoxia while leaving cytochrome *c* oxidase activity unchanged. In addition, we identify that glycolytic reprogramming plays a critical role in macrophage migration activity.

## Results

### Glucose metabolism in mildly hypoxic condition

**Figure 1:**
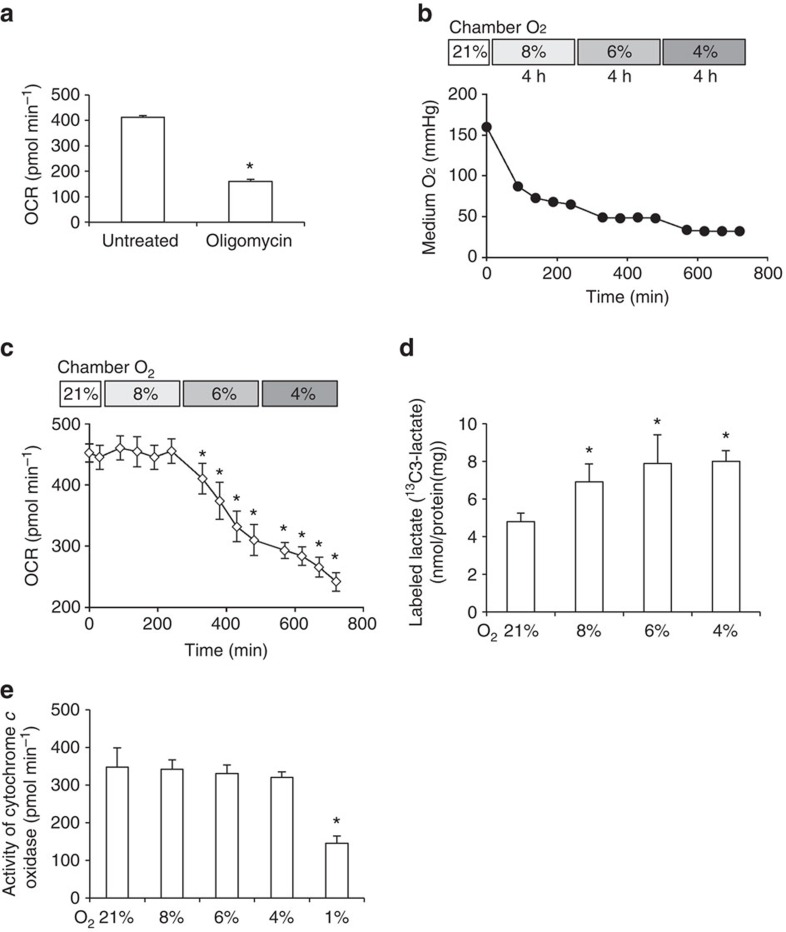
The glycolytic reprogramming of primary macrophages in mildly hypoxic condition. (**a**) The reduction of OCR by oligomycin (0.5 μM) to wild-type TEPMs. (**b**) The oxygen concentrations in assay medium were calibrated with XF24 Extracellular Flux Analyzer (8% O_2_, 4 h-6% O_2_, 4 h-4% O_2_, 4 h). (**c**) OCR of wild-type TEPMs was plotted as pmol min^−1^ (*y* axis) versus time (*x* axis; 8% O_2_, 4 h-6% O_2_, 4 h-4% O_2_, 4 h). (**d**) The lactate production in TEPMs was calculated with capillary electrophoresis (CE)-electrospray ionization (ESI)-mass spectrometry (MS; 8% O_2_, 4 h-6% O_2_, 4 h-4% O_2_, 4 h). *n*=5 animals. (**e**) The cytochrome *c* oxidase activity of TEPMs was calculated as OCR in the presence of ascorbate (100 mM) and TMPD (1 mM; 8% O_2_, 4 h-6% O_2_, 4 h-4% O_2_, 4 h-1% O_2_, 4 h). All graphs indicate average with s.d. All experiments using XF24 were repeated three times. TEPMs were collected from wild-type C57BL6 mice (**a**,**c**) and from *Tie2-Cre*^*−/−*^ *HIF-1α*^*flox/flox*^ mice (**d**,**e**). Student's *t*-test was performed to calculate *P* value. *P<0.05 versus control (21% O_2_); 8, 6, 4 and 1% O_2_ concentration, respectively.

To monitor the activity of mitochondrial-dependent glucose oxidation which is mediated by cytochrome *c* oxidase, we measured cellular oxygen consumption rate (OCR) with glucose as an energy substrate. In this study, we utilized murine primary peritoneal macrophages termed thioglycollate-elicited peritoneal macrophages (TEPMs). Oligomycin, an inhibitor of mitochondrial respiration, decreased 70% of OCR, which reflected the activity of glucose oxidation (Fig. 1a). We monitored OCR in normoxia (21% oxygen), followed by measurements every 4 h thereafter at decreasing oxygen concentrations for each time point. After calibrating the oxygen concentration in the culture medium (Fig. 1b), we measured the OCR at each O_2_ concentration. The OCR of TEPMs dropped at 6% O_2_, suggesting that an O_2_ concentration of >6% was required for the maintenance of glucose oxidation in TEPMs (Fig. 1c). Using capillary electrophoresis-mass spectrometry, we traced the fate of ^13^C-labelled glucose to monitor intracellular glucose metabolism in TEPMs. The output from ^13^C-labelled glucose to lactate significantly increased in mild hypoxia (6–8% oxygen; Fig. 1d), further supporting our finding that an O_2_ concentration of 6% was required for the maintenance of glucose oxidation by TEPMs. We next measured the activity of macrophage cytochrome *c* oxidase in the presence of ascorbate and TMPD, which ensures the cytochrome *c* oxidase to access a sufficient amount of electron input. While an O_2_ concentration of 1% led to a drop in cytochrome *c* oxidase activity, it remained almost unchanged at an O_2_ concentration of 4–8% (Fig. 1e). Although glucose oxidation was suppressed in mild hypoxia (4–6% oxygen), cytochrome *c* oxidase activity remained unaffected, implying the presence of another regulatory process in glucose oxidation in mild hypoxia.

### HIF-1α plays a critical role in glycolytic reprogramming

**Figure 2:**
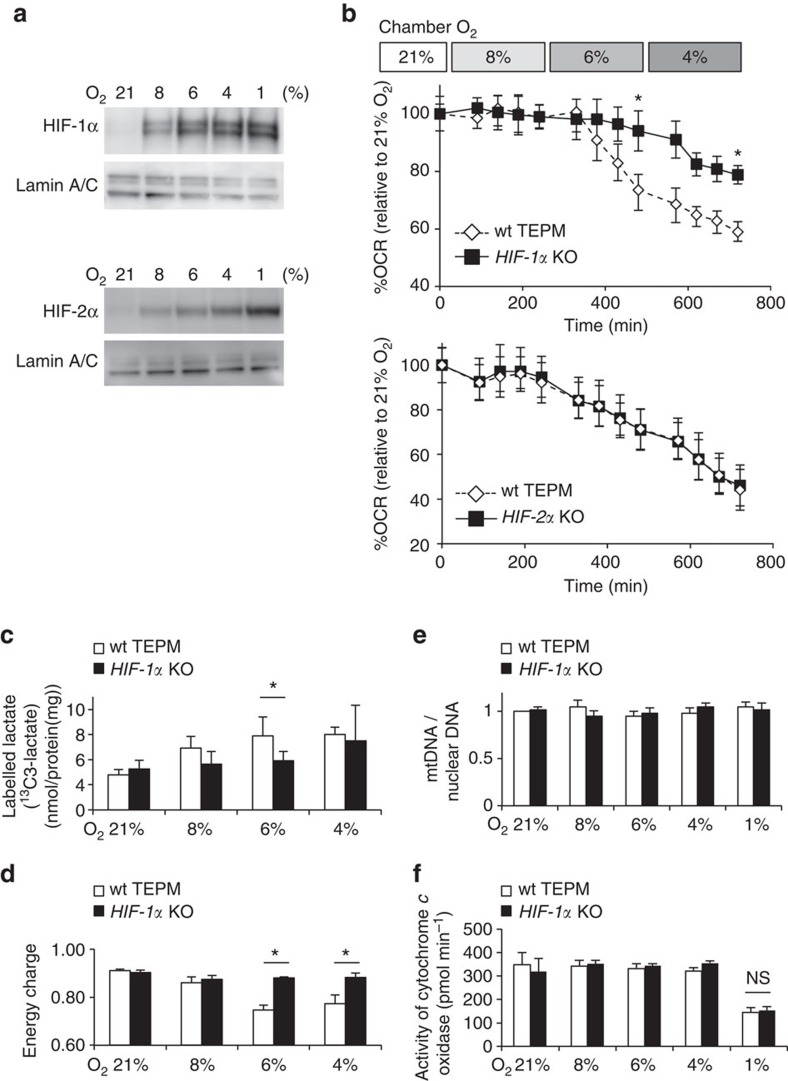
HIF-1α-dependent glycolytic reprogramming in mild hypoxia. (**a**) HIF-1α and HIF-2α protein abundance in wild-type TEPMs under each O_2_ concentration (21, 8, 6, 4 or 1% O_2_, 4 h) was determined by immunoblot analysis. Lamin A/C was used as a loading control. (**b**) OCR of TEPMs under mild hypoxia (8% O_2_, 4 h-6% O_2_, 4 h-4% O_2_, 4 h) was plotted as percent to OCR in 21% O_2_ (*y* axis) versus time (*x* axis). (**c**) The lactate production in hypoxic peritoneal macrophages was detected by using capillary electrophoresis (CE)-electrospray ionization (ESI)-mass spectrometry (MS). *n*=5 animals. (**d**) Energy charge was calculated with following formula: (ATP)+0.5 (ADP)/((ATP)+(ADP)+(AMP)). (**e**) Mitochondrial DNA (mtDNA) content measured relative to nuclear DNA in TEPMs under normoxia and hypoxia (21, 8, 6, 4 and 1% O_2_, 4 h). The results were shown relative to mtDNA/nuclear DNA in 21% O_2_. *n*=3 animals. (**f**) The cytochrome *c* oxidase activity of TEPMs was calculated as OCR in the presence of ascorbate (100 mM) and TMPD (1 mM; 8% O_2_, 4 h-6% O_2_, 4 h-4% O_2_, 4 h-1% O_2_, 4 h). All graphs indicate average with s.d. All experiments using XF24 were repeated three times. Student's *t*-test was performed to calculate *P* value. *P<0.05 wt TEPM versus *HIF-1α* KO; NS, not significant; wt TEPM, *Tie2-Cre*^*−/−*^ *HIF-1α*^*flox/flox*^ mice or *Tie2-Cre*^*−/−*^ *HIF-2α*^*flox/flox*^ mice; *HIF-1α* KO, *Tie2-Cre*^*+/−*^ *HIF-1α*^*flox/flox*^ mice; *HIF-2α* KO, *Tie2-Cre*^*+/−*^ *HIF-2α*^*flox/flox*^ mice; 21, 8, 6, 4, 1% O_2_ concentration, respectively.

Prolyl hydroxylase domain-containing proteins act as another oxygen sensor in the cytosol. In the presence of oxygen, prolyl hydroxylase domains hydroxylate and target HIF-1α and HIF-2α for proteasomal degradation^2^,^3^,^4^. HIF-α protein accumulates in hypoxia and induces the expression of hypoxia responsive genes^5^. We have analysed HIF-α isoform-specific function^6^,^7^,^8^, and identified that *Pdk1* as a HIF-1α target gene^9^,^10^. The results demonstrated that the accumulation of HIF-1α and HIF-2α proteins positively correlated with increasing levels of hypoxia (Fig. 2a). To examine the roles of HIF-1α or HIF-2α in glycolytic reprograming, we isolated TEPMs from haematopoietic/endothelial-specific HIF-1α-deficient mice (*HIF-1α*^*flox/flox*^*; Tie2-Cre*^*+/−*^*mice*; HIF-1α KO), HIF-2α-deficient mice (*HIF-2α*^*flox/flox*^*; Tie2-Cre*^*+/−*^*mice*; HIF-2α KO) or Cre recombinase-negative littermate controls. While the OCR of HIF-2α KO TEPMs dropped similar to wild-type TEPMs, the OCR of HIF-1α KO macrophages did not drop at 6% O_2_ (Fig. 2b). In addition, lactate output from ^13^C-labelled glucose was not elevated in HIF-1α KO TEPMs, suggesting that HIF-1α KO TEPMs could oxidize glucose under the condition of 6% O_2_ (Fig. 2c). Consistent with these results, the energy status calculated by energy charge of HIF-1α KO macrophages was higher than that of wild-type cells (Fig. 2d). The critical role of HIF-1α in glycolytic reprogramming in mild hypoxia was also observed in hepatocytes (Supplementary Fig. 1), supporting the notion that HIF-1α plays an essential role in the regulation of mitochondrial activity. Next, we evaluated mitochondrial activity in HIF-1α deficient cells. The mitochondrial DNA content was not altered between wild-type and HIF-1α-deficient macrophages (Fig. 2e). Moreover, cytochrome *c* oxidase activity in the presence of ascorbate and TMPD was not altered between wild-type and HIF-1α-deficient macrophages (Fig. 2f). The results showed that HIF-1α signal did not affect the maximum respiratory activity of the mitochondrial cytochrome *c* oxidase.

### HIF-1α-PDK1 axis regulates glycolytic reprogramming

**Figure 3:**
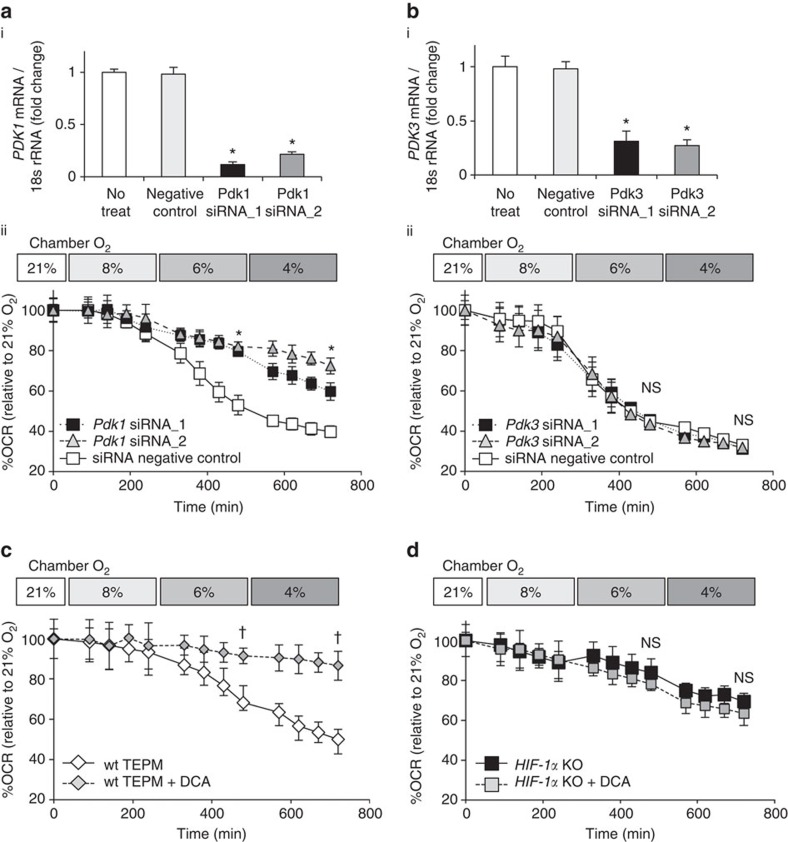
The essential role of HIF-1α - PDK1 axis in hypoxia-induced glycolytic reprogramming. (**a**,**b**) (i) The relative expression of *Pdk1 or Pdk3* in RAW 264.7 cells with gene silencing by siRNA. (ii) OCR of RAW 264.7 cells treated with Pdk1 or Pdk3 siRNA of under mild hypoxia (8% O_2_, 4 h-6% O_2_, 4 h-4% O_2_, 4 h). (**c**,**d**) OCR of TEPMs treated with DCA (10 mM), a chemical inhibitor of PDK, under mild hypoxia (8% O_2_, 4 h-6% O_2_, 4 h-4% O_2_, 4 h). All graphs indicate average with s.d. All experiments using XF24 were repeated three times. Student's *t*-test was performed to calculate *P* value. *P<0.05 versus control (negative control); ^†^*P*<0.05 versus control (untreated); NS, not significant; wt TEPM, *Tie2-Cre*^*−/−*^ *HIF-1α*^*flox/flox*^ mice; *HIF-1α* KO, *Tie2-Cre*^*+/−*^ *HIF-1α*^*flox/flox*^ mice; DCA ; 21, 8, 6 and 4% O_2_ concentration, respectively.

Pyruvate, a glucose metabolite, is dependent on the activity of pyruvate dehydrogenase complex to enter the tricarboxylic cycle. Each isozyme of PDK family phosphorylates and inhibits the function of pyruvate dehydrogenase complex, thus acting as a gatekeeper enzyme of glucose oxidation^11^. We and others have revealed that *Pdk1* or *Pdk3* expression increases in hypoxia through a HIF-1α-dependent manner^9^,^10^. To examine the roles of PDKs in glycolytic reprogramming, we adopted two different approaches, namely, RNA interference-mediated knockdown and chemical inhibition. To genetically manipulate the *Pdk1* or *Pdk3* gene, we utilized the macrophage-derived RAW 264.7 cell line. While knockdown of *Pdk3* did not affect the OCR in hypoxia, knockdown of *Pdk1* significantly restored the decline in OCR under hypoxic conditions (Fig. 3a,b). We next tested the effect of dichloroacetic acid (DCA), a chemical inhibitor of PDK, on glucose metabolism. While DCA significantly prevented the decline in OCR under hypoxic conditions, it had almost no effect on the OCR of HIF-1α-deficient macrophages (Fig. 3c,d). These results imply that HIF-1α-PDK1 axis actively induces glycolytic reprograming in mildly hypoxic condition through the redirection of pyruvate flux into lactate.

Under hypoxic conditions, the HIF-1α protein accumulates and binds to hypoxia responsive elements (HREs), thereby activating the transcription of its target genes^5^. The expression of several glycolytic genes, such as *glucose transporter-1* (*Glut1*), *phosphoglycerate kinase 1* (*Pgk1*) or *lactate dehydrogenase A* (*Ldha*), was significantly induced in mild hypoxia through a HIF-1α, but not HIF-2α-dependent pathway (Supplementary Fig. 2a,b). Intriguingly, *Pdk1* expression was the most rapidly induced under hypoxic conditions. The expression of other *Pdk* isozymes, *Pdk2*, *Pdk3* and *Pdk4* were either unchanged or undetected in primary macrophages.

To investigate whether the HIF-1α binding affinity to the HRE of the *Pdk1* gene was different from that of other hypoxia-induced genes, we examined the binding of HIF-1α to promoter/enhancer regions of its target genes^12^. Additionally, it revealed that HIF-1α not only binds to the promoter region of *Pdk1* but to other glycolytic genes under mildly hypoxic conditions. The results suggest that HIF-1α binding affinity to the HRE of *Pdk1* is unaltered when compared with that of other HIF-1α target genes (Supplementary Fig. 2c). In addition to the rate of transcription of each of the target genes, the stability of each transcript strongly affects its abundance^13^. Therefore, we checked the messenger RNA (mRNA) stability of each hypoxia-inducible gene by inhibi.ting their transcription with actinomycin D. Among the HIF-1α target genes we examined, the *Pdk1* transcript had the lowest stability, suggesting that *Pdk1* mRNA possesses the highest turnover rate among the hypoxia-inducible genes (Supplementary Fig. 2d, Supplementary Table 1). Thus, the high turnover rate of the *Pdk1* mRNA may account for its rapid induction in mild hypoxia.

### The role of glycolysis in macrophage migratory capacity

**Figure 4:**
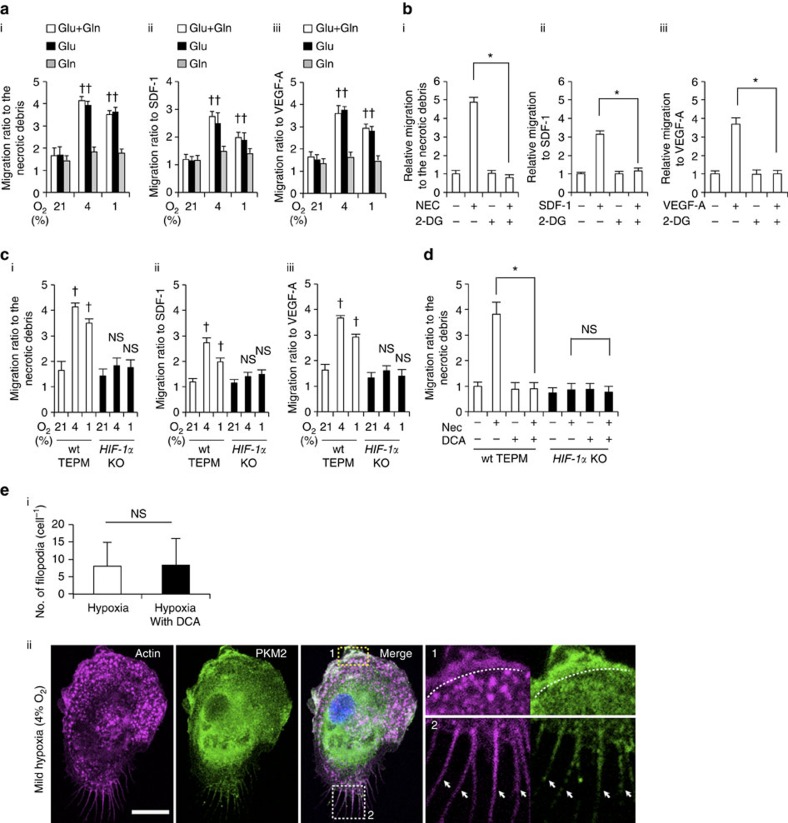
The role of glycolytic reprogramming in macrophage migratory capacity *in vitro*. (**a**) (i-iii) Macrophage migration activity to various chemoattractants (the supernatant of necrotic cell debris (i), stromal cell-derived factor 1 (SDF-1; 100 ng ml^−1^; ii) and VEGF-A (100 ng ml^−1^; iii)) was measured by transwell assay in normoxia (21% O_2_, 4 h), mild hypoxia (4% O_2_, 4 h) and severe hypoxia (1% O_2_, 4 h). The culture mediums were supplemented with 10 mM glucose (Glu) only, 2 mM L-glutamine (Gln) only or both. The *y* axis represents the migration activity relative to untreated condition. *n*=3 animals. (**b**) Macrophage migration activity was measured in mild hypoxia in the presence of 2-DG (20 mM). *n*=3 animals. (**c**) Migration activity in wild-type and HIF-1α-deficient macrophages was measured in the same manner as 4 (**a**). *n*=3 animals per group. (**d**) Macrophage migration activity to the supernatant of necrotic cell debris was measured in mild hypoxia in the presence of DCA (10 mM). *n*=3 animals per group. (**e**) (i) The number of filopodia in TEPMs is shown. TEPMs were cultured under mild hypoxia in the presence or absence of DCA (10 mM). (average with s.d. *n*=100 cells analysed). (ii) Representative immunocytochemical staining of Hoechst (blue), PKM2 (green) and Phalloidin (magenta) in TEPMs under mild hypoxia is shown. F-actin and PKM2 were co-localized in lamellipodia (magnified view 1, dotted line) and filopodia (magnified view 2, arrowheads). Scale bars, 10 μm. All graphs indicate average with s.d.. Student's *t*-test was performed to calculate *P* value. *P<0.05; NS, not significant; ^†^*P*<0.05 versus control (21% O_2_); 2-DG, 2-Deoxy-D-glucose; NEC, the supernatant of necrotic cell debris.

Migration and activation are the two key functions of macrophages that promote inflammatory processes^7^,^14^. During the process of inflammation, monocyte-derived macrophages extravasate from blood vessels and migrate to the site of tissue injury or infection within a few hours^15^. While intravascular oxygen content remains high, local oxygen concentrations in inflammatory areas are believed to be low^16^,^17^, causing monocyte-derived macrophages to encounter a gradual decrease in oxygen availability during the migration process in inflammatory areas^18^. Therefore, we examined the roles of active glycolytic reprogramming in macrophage migration activity. The chemokines stromal cell-derived factor 1 or vascular endothelial growth factor-A acts to induce chemotaxis in primary macrophages^19^,^20^. In addition, the supernatant of necrotic cell debris is known to be a chemoattractant for the macrophages^21^. We measured macrophage mobilization towards these chemoattractants in normoxia (21% oxygen), mild hypoxia (4% oxygen) and severe hypoxia (1% oxygen). Macrophages migrated more rigorously to these chemoattractants in mild hypoxia than in normoxia (Fig. 4a). TEPM mobilization activity to some chemokines was equivalent to or even higher in mild hypoxia compared with that of severe hypoxia. Intriguingly, macrophage migratory activity was significantly decreased in glucose depleted conditions (Fig. 4a). Strikingly, the inhibition of glucose uptake by 2-deoxy-D-glucose suppressed macrophage migration, thereby illuminating a role for glucose metabolism in macrophage mobilization (Fig. 4b). The expression of chemokine receptors, *Flt-1* or *Cxcr4* was unaffected in mild hypoxia, further supported the roles of macrophage metabolism in cellular migration (Supplementary Fig. 3a,b). Hypoxia-induced acceleration of macrophage migration was absent in HIF-1α-deficient macrophages, suggesting that HIF-1α signalling is required for macrophage migratory capacity (Fig. 4c). On the other hand, HIF-2α signalling was dispensable for macrophage migratory activity (Supplementary Fig. 4). To examine whether macrophage migration depends on glucose oxidation or glycolysis, DCA was used to inhibit macrophage glycolysis. Intriguingly, DCA strongly suppressed the migration of primary macrophages *in vitro*, suggesting that glycolytic activity is required for macrophage mobilization (Fig. 4d). Neither inhibition of glucose uptake nor glycolysis affected the migratory activity of HIF-1α-deficient macrophage. To further investigate the link between glycolytic metabolism and macrophage migration, we tested the effect of DCA on the macrophage cytoskeletal structure. We performed F-actin staining with fluorescently labelled phalloidin; however, we did not find a striking difference in the number of filopodia between control and DCA-treated macrophages (Fig. 4e). Cytosolic ATP is rapidly consumed during cell migration processes^22^. While migratory capacity of HIF-1α deficient macrophages is decreased, its total energy charge is higher than that in wild-type macrophages (Fig. 2d). Therefore, we next examined the intracellular localization of glycolytic enzymes in TEPMs. A glycolytic enzyme, pyruvate kinase muscle (PKM2) is responsible for ATP synthesis and plays an essential role in macrophage activation^23^. Immunocytochemical analysis revealed that PKM2 is co-localized with F-actin in filopodia and lamellipodia (Fig. 4e), which is consistent with the recent reports showing the intracellular localization of glycolytic enzymes in vascular endothelial cells or erythrocytes^24^,^25^,^26^. These results imply the critical role of glycolytic metabolism in macrophage migration.

### DCA suppresses macrophage migration *in vivo*

**Figure 5:**
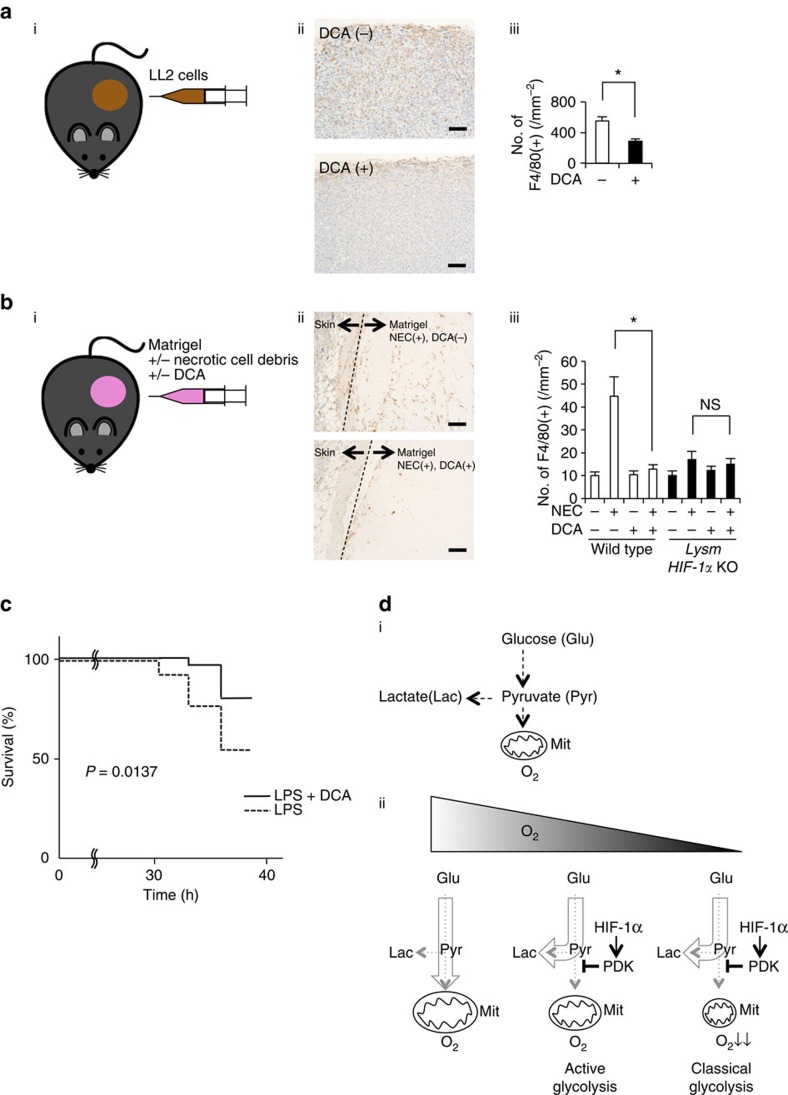
DCA suppresses macrophage migration *in vivo*. (**a**) (i) LL2 cells (1 × 10^6^) were subctaneously injected in wild-type mice. (ii) Representative immunohistochemical staining of F4/80 (brown) in the isolated tumor from mice with or without DCA treatment (1.5 g l^−1^ in drinking water for 8 days). (iii) The *y* axis of the graph represents the number of F4/80-positive stained macrophages per field (10 random fields per tumor). *n*=7 animals per group. (**b**) (i) Matrigel (500 μl) containing the supernatant of necrotic cell debris with or without DCA (10 mM) was subctaneously injected in wild-type and *LysM HIF-1α* KO mice. (ii) Representative immunohistochemical staining of F4/80 (brown) in the isolated matrigel in the presence or absence of DCA. (iii) The *y* axis represents the number of F4/80-positive macrophages per field (10 random fields per matrigel) in wild-type and *LysM HIF-1α* KO mice. *n*=5 animals per group. (**c**) Mice were pre-treated with DCA (100 mg kg^−1^ body weight) or PBS for 6 h (*n*=50 versus 50). Subsequently, endotoxin shock was induced by the intraperitoneal injection of lipopolysaccharide (LPS; Sigma; 40 mg kg^−1^ body weight). (**d**) (i) The schema of glucose metabolism. (ii) The schematic representation of classical and active glycolysis in hypoxia. All graphs indicate average with s.d. Student's *t*-test was performed to calculate *P* value. *P<0.05; NS, not significant; wild-type, *LysM-Cre*^*−/−*^ *HIF-1α*^*flox/flox*^ mice; *LysM HIF-1α* KO, *LysM-Cre*^*+/−*^ *HIF-1α*^*flox/flox*^ mice; NEC, the supernatant of necrotic cell debris; Scale bar, 100 μm.

To further examine the role of hypoxia in macrophage mobilization *in vivo*, we performed tumour xenograft implantation model using Lewis lung carcinoma (LL2) cells (Fig. 5a), which is known to elicit hypoxic condition in tumour tissues. As reported previously, macrophages accumulated in a severely hypoxic area in tumour tissue (Supplementary Fig. 5)^17^. Using LL2 xenograft model, we evaluated the effect of DCA on macrophage accumulation to the hypoxic area *in vivo*. Because DCA potentially affects tumour growth^27^,^28^, we measured macrophage accumulation 8 days after LL2 implantation, when tumour sizes of control or DCA-treated groups were almost equivalent. Intriguingly, DCA strikingly suppressed macrophage accumulation in tumour tissue (Fig. 5a).

To evaluate directly the effect of DCA on macrophage migration *in vivo*, we implanted matrigel containing necrotic cell debris subcutaneously, and observed the macrophage mobilization (Fig. 5b). While blood vessels were scarcely found in the matrigel, one of the hypoxia-inducible gene, *carbonic anhydrase IX* (CA9), was highly expressed in the macrophages inside the gel (Supplementary Fig. 6). These results indicate that the tissue inside matrigel was in hypoxic state. Notably, DCA significantly inhibited the migration of macrophages to matrigels containing the supernatant of necrotic cell debris. To further test the roles of HIF-1α on macrophage migration *in vivo,* we generated myeloid-cell-specific HIF-1α-deficient mice (*HIF-1α*^*flox/flox*^*; LysMCre*^*+/−*^, *LysM HIF-1α* KO)^6^. While macrophage recruitment was significantly suppressed in *LysM HIF-1α* KO mice, DCA did not affect macrophage mobilization in these mice (Fig. 5b). These results indicate that inhibition of HIF-1α-PDK1 axis significantly suppresses macrophage migration in hypoxic conditions.

We and others previously reported that HIF-1α deletion in macrophages was protective against an inflammation induced by lipopolysaccharide^7^,^29^. To investigate the role of glycolytic reprogramming in systemic inflammation, we examined the effect of PDK inhibition on the severity of sepsis. DCA significantly suppressed the inflammatory gene expression such as *IL-1β* in macrophages (Supplementary Fig. 7). Moreover, pretreatment with DCA significantly ameliorated the survival or severity of the shock status induced by lipopolysaccharide injection (Fig. 5c). While lipopolysaccharide induced systemic inflammation was attenuated in *LysM HIF-1α* KO mice, DCA had no further effect in these HIF-1α deficient mice (Supplementary Fig. 8).

Collectively, the data suggests that HIF-1α-PDK1 axis induced active glycolysis underlies the macrophage migration and activation processes that take place during the systemic inflammation.

The Warburg effect refers to the condition under which a cancer cell undergoes glycolysis even in the presence of oxygen. In contrast, the glucose metabolism of non-cancer cells flexibly changes depending on the availability of molecular oxygen. A phenomenon referred to as the Pasteur effect^30^. While mitochondrial electron transport chain is essential in glucose oxidation, the activity of cytochrome *c* oxidase is suppressed under severely hypoxic conditions, resulting in a metabolic shift towards glycolysis (classical glycolysis). Previously, we demonstrated that HIF-1α-mediated activation of glycolysis had a beneficial effect for the synthesis of ATP in severely hypoxic conditions^31^. Here, we reported a novel glycolytic process which takes place in mildly hypoxic conditions. HIF-1α-PDK1 axis actively changes the glucose metabolism from glucose oxidation into glycolysis, which in turn decreases cellular ATP level, showing a two-sided role of HIF-1α in ATP synthesis. HIF-1α-induced PDK1 sequesters glucose metabolite flux from the mitochondria to glycolysis. This glycolytic shift can be referred to as active glycolysis because it redirects the metabolic flux irrespective of the mitochondrial activity in contrast to the classical glycolysis in severe hypoxia (Fig. 5d). Active glycolysis occurred not only in primary macrophages but also in primary hepatocytes, suggesting it may be a common feature of glucose metabolism in non-cancer cells.

Active glycolysis in each non-cancer cell may play an essential role in cell-type-specific function through the alteration of intracellular ATP distribution. In this study, we revealed that glycolytic reprogramming in hypoxia plays an essential role in macrophage mobilization, which agreed with previous reports that shed light on the link between glycolysis and cytoskeletal actin remodelling^24^,^25^,^26^. During cell migration processes, ATP is rapidly consumed in the cytosol for the remodelling of cytoskeletal actin filaments^32^. Therefore, it has been proposed that synthesis of ATP in proximity with cytosolic F-actin is beneficial to meet the demand of ATP during cell migration processes^25^,^33^. Our results agreed with this hypothesis in that glycolytic reprogramming, not glucose oxidation, plays a crucial role in the process of macrophage migration. However, it still remains to be elucidated why HIF-1α-deficient macrophages exhibit reduced migratory activity, whereas total ATP level in HIF-1α deficient macrophage was higher than that in wild-type macrophages. In addition, active glycolysis may be beneficial in preventing excessive amounts of reactive oxygen species production, which is elicited when mitochondrial respiration is damaged.

While several inflammatory and glycolytic genes are known to be targets of HIF-1α, the temporal profile of their induction has not been fully elucidated. We found that the expression of *Pdk1* gene was more rapidly induced in hypoxia than *iNOS* or *Glut1*. The accumulation of HIF-1α at the HRE region was not altered, whereas the mRNA turnover kinetics for each gene was quite different. The abundance of mRNA is regulated through the balance between its synthesis and degradation^13^. Remarkably, we identified the transcript of *Pdk1* had a significantly shorter half-life than that of *iNOS* or *Glut1*, which accounts for the rapid induction of *Pdk1* gene in hypoxia. These results uncovered the previously undescribed effects of mRNA stability in the dynamic of hypoxic gene induction.

Accumulating evidence discloses the roles of metabolic reprogramming in cytokine production^23^,^29^. The results in this study have also uncovered a novel link between hypoxia-elicited metabolic reprogramming and macrophage migratory capacity. Infiltration of pro-inflammatory cells into areas of inflammation plays an essential role during the pathogenesis of sepsis, arthritis or atherosclerosis. Modulation of macrophage intracellular metabolism, therefore, could potentially be a therapeutic target in regulating the severity of several inflammatory diseases.

## Methods

### Mice

All animal experiments were approved by the University of Tokyo Ethics Committee for Animal Experiments and the University of Texas at Dallas Institutional Animal Care and Use Committees, and strictly adhered to the animal experiment guidelines. Mice carrying the *lox*P—flanked conditional alleles of HIF-1α in C57BL/6 background were used those we generated previously^34^. HIF-2α floxed mice were obtained from Jackson Laboratories (Bar Harbor, ME). These mice were bred with *Tie2-Cre* (ref. 35), *LysM-Cre* (ref. 36) or *Alb-Cre* (ref. 37) mice to generate mice with haematopoietic/endothelial-specific, myeloid-specific or hepatocyte-specific deletion of the HIF-1α gene (HIF-1α KO) or the HIF-2α gene (HIF-2α KO) on a C57BL/6 background. Total RNA was isolated from TEPMs of the mice, and efficacy of deletion was calculated by quantitative PCR with primers spanning the target region as well as primers for an undeleted control gene for normalization (Supplementary Fig. 9). Cre-negative homozygous littermates for the conditional alleles were used as controls. Female mice 7–10 weeks of age were used for all experiments.

### Cell culture

TEPMs were isolated from the peritoneal cavity 4 days after the intraperitoneal injection of a 3% thioglycollate solution (Fluka, Sigma-Aldrich, St Louis, MO, USA). TEPMs, the murine-derived macrophage cell line RAW 264.7 cells and LL2 (ATCC, Manassas, VA, USA) were cultured using RPMI 1640 (GIBCO, Life technologies, Carlsbad, CA, USA) supplemented with 1% (v/v) penicillin/streptomycin (Sigma-Aldrich), if not otherwise specified. Hepatocytes were isolated from mice by digesting the liver with type IV collagenase^38^,^39^. All perfusions were carried out at flow rates of 4 ml min^−1^ and all buffers were maintained at 37 °C. The first buffer was made up of Hank's-HEPES solution containing 1 mM EGTA, and the second buffer was made up of Hank's-HEPES containing collagenase I (0.5 mg ml^−1^; Wako, Osaka, Japan). Hepatocytes were purified by washing twice with ice-cold Hank's-HEPES buffer solution followed by Percoll gradient separation. Hepatocytes were suspended in complete culture medium (William's medium E containing 10% foetal bovine serum, 0.5 nM insulin, 100 nM dexamethasone and EGF, 20 ng ml^−1^). All cells were cultured at 37 °C in a humidified CO_2_ incubator. Exposure of cell cultures to hypoxia (8%-1% O_2_) was undertaken in the hypoxic workstation *In Vivo*_2_ 500 (Ruskinn Technology, Bridgend, UK).

### Measurement of oxygen consumption rate

A XF24 Extracellular Flux Analyser (Seahorse Biosciences, North Billerica, MA, USA) was used to determine the intracellular bioenergetic profiles. The cell numbers seeded onto each well of a XF24 V7 Cell Culture microplate were as follows: 1 × 10^5^ for TEPMs, 1 × 10^5^ for RAW 264.7 cells and 5 × 10^4^ for hepatocytes. OCR was assessed in glucose-containing XF base medium according to the manufacturer's instructions. The schedule of the gradual hypoxia assay is shown in Fig. 3a. The experiment examined the sequential electron flow through different complexes of the electron transport chain was performed by sequential addition of antimycin A (4 mM) and ascorbate/tetramethyl-p-phenylenediamine (TMPD) (1 mM and 100 mM, respectively)^40^, using the XF Plasma Membrane Permeabilizer (Seahorse Biosciences). Oligomycin and dichloroacetate were obtained from Sigma for additional treatments.

### Quantitative RT-PCR

For gene expression analysis, total RNA was purified from cultured cells using RNeasy kits (Qiagen, Tokyo, Japan) according to the manufacturer's instructions. Complementary DNA was synthesized using the SuperScript III First-Strand Synthesis System (Life Technologies). Quantitative real-time PCR (RT-PCR) analyses were conducted using the LightCycler system (Roche Diagnostic, Tokyo, Japan), with *18S* rRNA serving as the internal control. For the mRNA stability assay, cells were treated with 5 μM actinomycin D (Sigma-Aldrich) to block their mRNA synthesis at each time point, and gene expressions of time-course samples were examined^8^. For the quantitative estimation of nuclear and mitochondrial DNA, total DNA was purified by QIAamp DNA Blood Mini Kit (Qiagen). Primer sequences of the analysed mouse genes are shown in Supplementary Table 2. The relative expression under each condition was normalized to the sum of all the conditions.

### Cell fractionation and immunoblotting

Cytoplasmic and nuclear extracts under normoxic or hypoxic conditions were prepared using the NE-PER Nuclear and Cytoplasmic Extraction Kit (Thermo Scientific, Rockford, IL, USA). Soluble fractions were prepared by the sampling buffer (20 mM Tris, pH 7.4, 150 mM NaCl, 1 mM EDTA, 1 mM EGTA, 1% Triton X-100, 1x Protease Inhibitor Cocktail, 1x Phosphatase Inhibitor Cocktail)^41^. Ten micrograms of nuclear protein were separated on 4–8% Tris-Acetate gels (Thermo Scientific), and then transferred to polyvinylidene difluoride membrane (Millipore, Bedford, MA, USA). After blocking in 5% dry milk in Tris-buffered saline with Tween 20, the membrane was incubated with primary antibodies followed by incubation with horseradish peroxidase-conjugated secondary antibodies. Detection was performed using ECL Prime (GE Healthcare, Logan, UT, USA) according to the manufacturer's instructions. The following antibodies were used: HIF-1α (NB100-449, Novus Biologicals, Littleton, CO, USA), HIF-2α (AF2997, R&D systems, NE, USA), Lamin A/C (2032S, Cell Signaling Technology, Danvers, MA, USA), goat anti-rabbit IgG-HRP (sc-2004, Santa Cruz Biotechnology, Santa Cruz, CA, USA), Flt-1 (ab9540, Abcam, Camgridge, UK), CXCR4 (66042, Proteintech, Illinois, USA). The non-cropped blots of the representative images are shown in Supplementary Fig. 10. This experiment was successfully repeated three times.

### siRNA-mediated PDK1 and PDK3 knockdown

siRNA for murine PDK1, PDK3 and a universal negative control siRNA were purchased from Life Technologies. RAW 264.7 cells were transfected with these siRNAs using Lipofectamine 2000 (Life Technologies) according to the manufacturer's instructions. The media was replaced after transfection, and the knockdown efficiency was assessed by quantitative RT-PCR.

### ChIP assay

Chromatin immunoprecipitation (ChIP) was performed according to previous reports^9^,^42^. Cells were cross-linked for 10 min using 1% paraformaldehyde and then subjected to fragmentation using Sonifier (Branson, Dansbury, USA). Samples were immunoprecipitated with the HIF-1α antibody (NB100-134, Novus Biologicals). ChIP samples were also quantified with RT-PCR using specific primer pairs. All mouse primer sequences for PCR are listed in Supplementary Table 2.

### Capillary electrophoresis–electrospray ionization–mass spectrometry

A detailed Methods section describing all procedures and protocols is available in the Supplementary Method.

### *In vitro* and *in vivo* migration assay

*In vitro* migration assays were performed using Boyden chambers^43^. TEPMs treated with Calcein AM (C393, DOJINDO, Kumamoto, Japan) were plated onto HTS FluoroBlok inserts with 8-μm pores (BD, Falcon, San Jose, CA, USA) in serum-free RPMI 1640. C2C12 necrosis was induced by H_2_O treatment for 1 h at 37 °C (ref. 21). Media conditioned with the supernatant of necrotic cell debris of C2C12 (ATCC, Manassas, VA, USA), stromal cell-derived factor 1 or VEGF-A (Sigma-Aldrich) was added to the lower wells of Boyden chambers^44^, and migrating cells were observed using the EVOS cell imaging system (Life Technologies). *In vivo* migration assays were performed with Matrigel GFR (BD, Falcon)^45^. Briefly, mice were subcutaneously injected with 500 μl of Matrigel containing either 50 μl of PBS or the supernatant of necrotic cell debris of the C2C12 cell line. Four days after matrigel implatation, skin tissues were removed and stained with carbonic anhydrase-IX (CA9; NB100-147, Novus Biological) and F4/80 (MCA497, AbD Serotec, Kidlington, UK). Macrophage recruitment was evaluated by counting the number of F4/80-positive cells.

### LL2 tumour xenograft assay

LL2 cells (1 × 10^6^) were injected subcutaneously in the left flank of wild-type mice. DCA was added in the drinking water of the treatment group (1.5 g l^−1^). Eight days after LL2 cells implantation, mice were killed and tumours were removed and stained with Pimonidazole (Cosmobio, Tokyo, Japan) and F4/80 (MCA497, AbD Serotec). Macrophage recruitment was evaluated by counting the number of F4/80-positive cells.

### Endotoxin shock

Lipopolysaccharide challenge was performed according to previous reports^46^,^47^. Mice were pre-treated with DCA (100 mg kg^−1^ body weight) or PBS for 6 h, after which endotoxin shock was induced by the intraperitoneal injection of lipopolysaccharide (Sigma; 40 mg kg^−1^ body weight). Mice were closely observed and euthanized quickly, at a humane end point (no locomotion, signs of severe diarrhoea or body weight loss exceeding 30% of the initial body weight). The mice were followed until the survival rate of either one of the groups fell to below 50%.

### Immunocytochemistry

Wild-type TEPMs were collected 4 days after intraperitoneal injection of 4% thioglycollate solution and were plated onto a non-coated 8-well chamber slide (2.5 × 10^4^ cells per well; Matsunami). Non-adherent cells were removed after 3 h and adherent macrophages were cultured in RPMI medium (Invitrogen) containing 10% FBS (JRH Biosciences) and 50 μg ml^−1^ penicillin/streptomycin (Wako) under atmospheric O2 condition. The cells were transferred to mild hypoxia (4% O_2_, 4 h) 1 day after plating and were cultured for another 13–16 h with or without 10 mM DCA. Cells were fixed with 4% paraformaldehyde (PFA) at 37 °C for 5 min, washed and processed for immunostaining with the following antibodies and reagent; anti-PKM2 antibody (x 1/100; CST; #4053; Rb mAb), anti-Rb-Alexa 488 (x 1/500; Invitrogen), Hochest (4 μM), phalloidin conjugated with Rhodamine or Alexa-633 Fluor(x 1/100; Invitrogen). Images were acquired using a SP8 inverted confocal microscopy (TCS SP8; Leica) with a × 63 oil objective lens (HCX PL APO 63 × /1.40–0.60 oil CS lens; Leica) and HyD detectors (Leica).

### Statistical analysis

All data are shown as means±s.d. Differences between two groups were analysed using Student's *t*-test. *P* values of <0.05 were considered to be statistically significant. The survival curves were calculated by the Kaplan–Meier method and compared by log-rank test. All statistical analyses were performed using SPSS software (IBM, Armonk, NY, USA).

### Data availability

The authors declare that the data supporting the findings of this study are available within the article.

## Additional information

**How to cite this article:** Semba, H. *et al.* HIF-1α-PDK1 axis-induced active glycolysis plays an essential role in macrophage migratory capacity. *Nat. Commun.* 7:11635 doi: 10.1038/ncomms11635 (2016).

## Supplementary Material

Supplementary Information Supplementary Figures 1-10 and Supplementary Tables 1-3
