# Supplementary material for: HIF-1α-PDK1 axis-induced active glycolysis plays an essential role in macrophage migratory capacity
Source: Nat Commun. 2016 May 18;7:11635. doi: 10.1038/ncomms11635 (PMC4873978; doi:10.1038/ncomms11635)
Supplement: Supplementary Information — Supplementary Figures 1-10 and Supplementary Tables 1-3 [file ncomms11635-s1.pdf]

Supplementary Figures

Figure-S1 (Takeda)

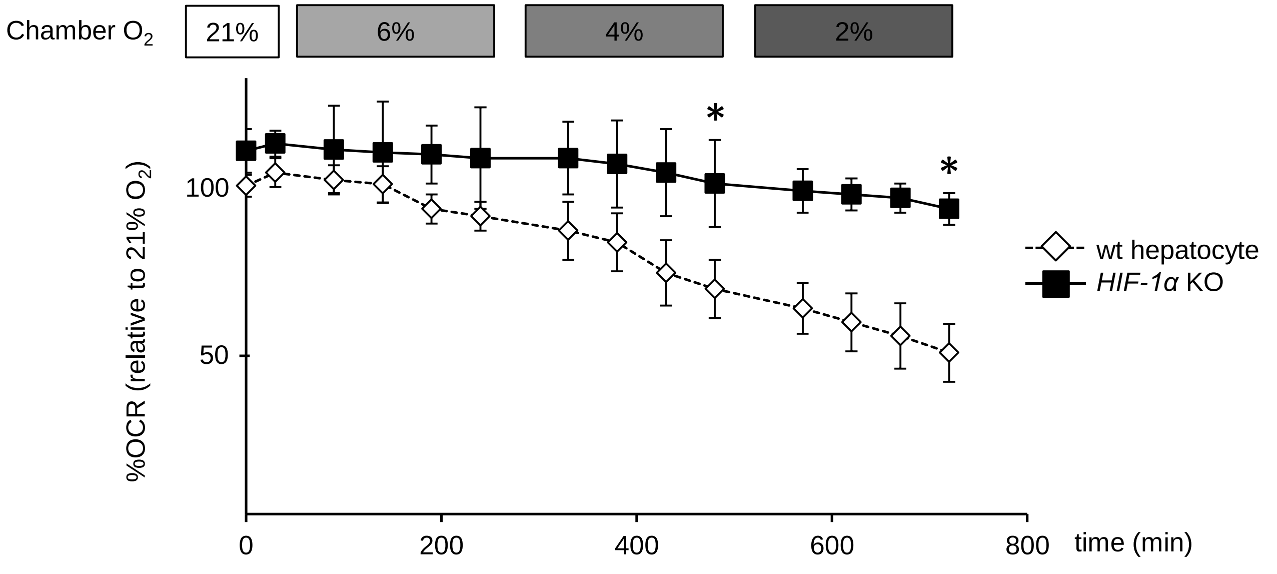

Hypoxia-induced glycolytic reprogramming was observed in hepatocytes. The ratio of oxygen consumption rate (%OCR) of hepatocyte in mild hypoxia was plotted as % relative to 21% O_2 (y-axis) vs. time (x-axis). wt hepatocyte, *Alb-Cre*$^{-/-}$ *HIF-1α*$^{flox/flox}$ mice; *HIF-1α* KO, *Alb-Cre*$^{+/-}$ *HIF-1α*$^{flox/flox}$ mice; *, $P < 0.05$ wt hepatocyte versus *HIF-1α* KO; 21%, 8%, 6%, 4%, O_2 concentration, respectively.

Figure-S2 (Takeda)

a

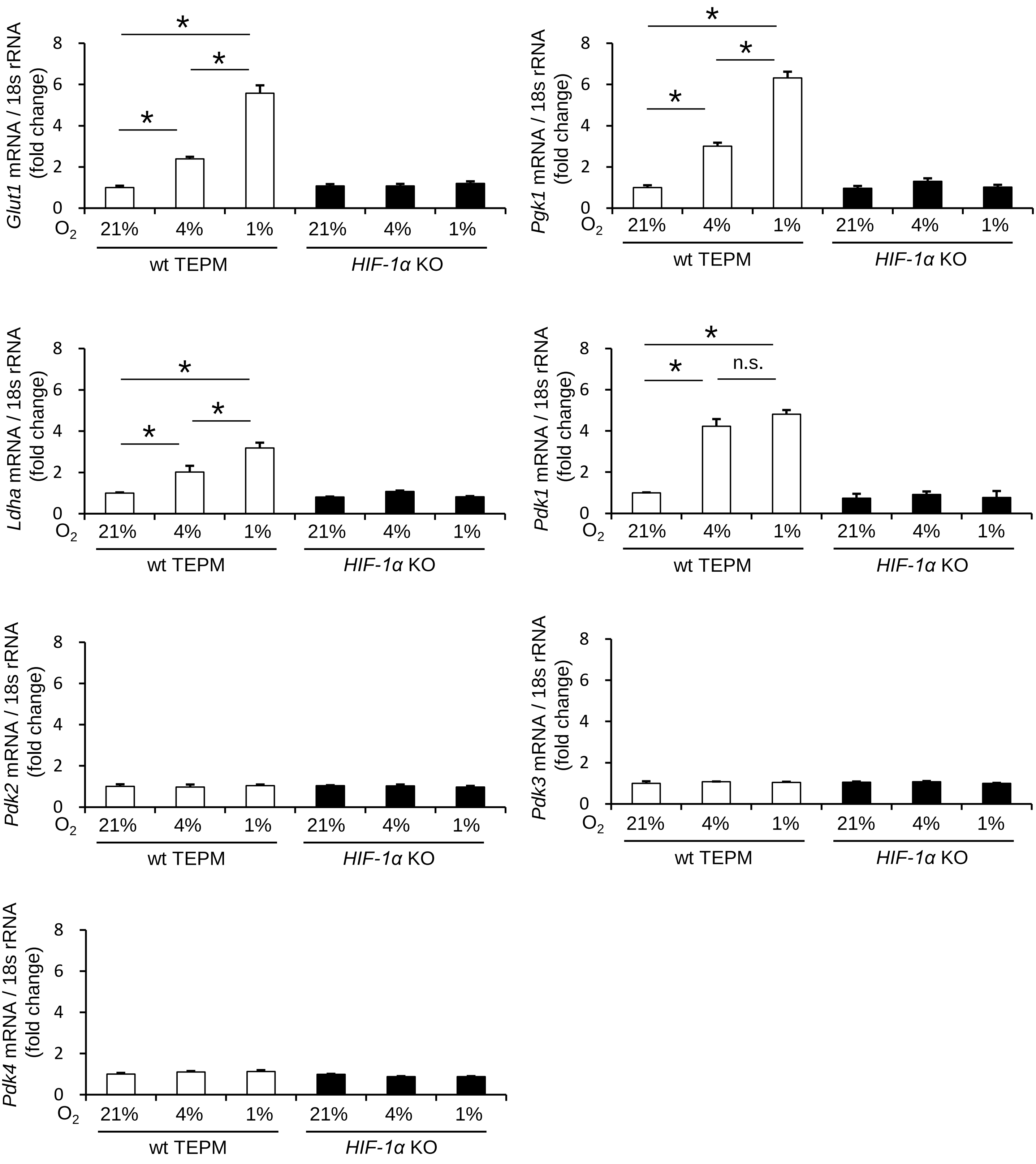

Figure-S2 (Takeda)

b

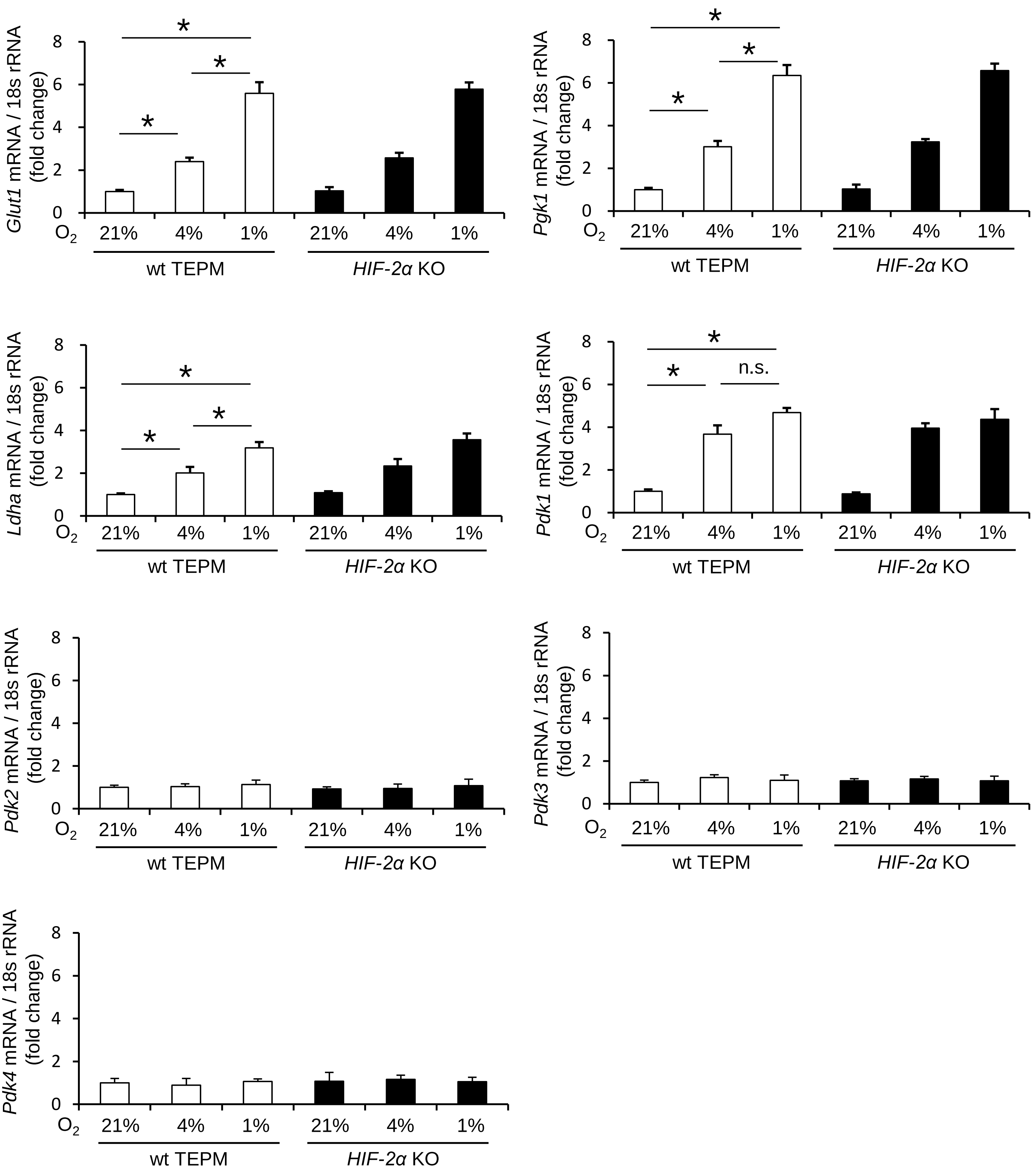

Figure-S2 (Takeda)

c

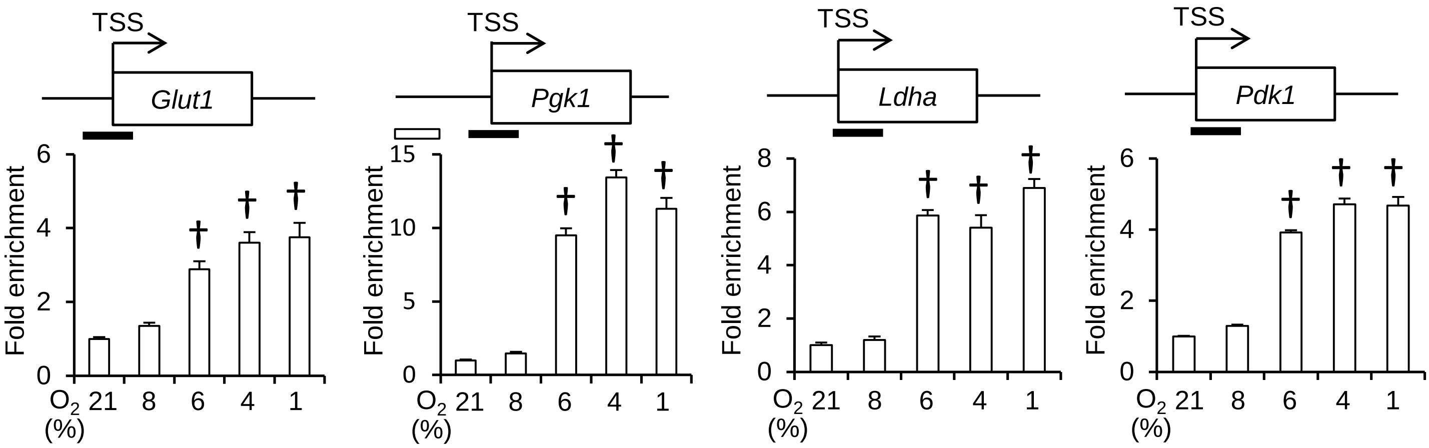

d

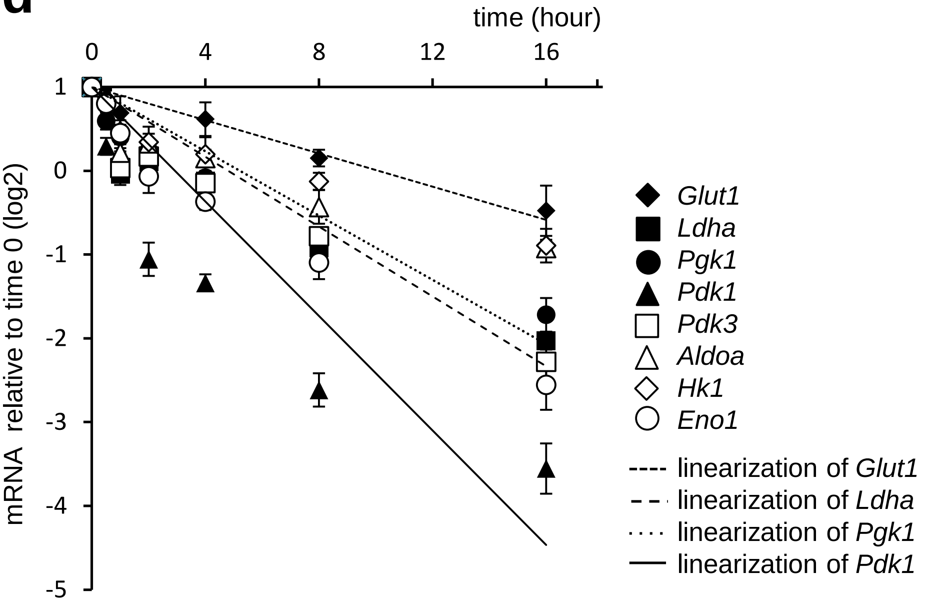

(a)(b) The gene expressions of glycolytic enzymes in normoxia (21 % O_2, 4h), mild hypoxia (4 % O_2, 4h) and severe hypoxia (1 % O_2, 4h) were assessed by quantitative RT-PCR. n = 3 animals.
(c) ChIP PCR analysis of HIF-1α binding to HRE. Black bar indicates the region amplified with quantitative RT-PCR in the schemas and represents the location of the Hypoxia Responsive Elements (HRE) in the promoter / enhancer of the HIF-1α target gene (See Supplementary Table 2 for sequence). White bar indicates the upstream region of *Pgk1* as a negative control, the region without HRE. Real-time PCR quantification of HIF-1α binding to these HREs is indicated as the relative enrichment compared to the binding to the negative control region. n = 3 animals.
(d) Wild-type TEPMs were treated with actinomycin D (5 mM) and the total RNA was collected every 4hr up to 16hr. The mRNA abundances of HIF-1α target genes are shown as relative to time 0. n = 3 animals.
All graphs indicate average with SD. *, P < 0.05; n.s., not significant; †, P < 0.05 vs control (21% O_2);
wt TEPM in (a), *Tie2-Cre*$^{-/-}$ *HIF-1α*$^{flox/flox}$ mice; *HIF-1α* KO in (a), *Tie2-Cre*$^{+/-}$ *HIF-1α*$^{flox/flox}$ mice;
wt TEPM in (b), *Tie2-Cre*$^{-/-}$ *HIF-2α*$^{flox/flox}$ mice; *HIF-1α* KO in (a), *Tie2-Cre*$^{+/-}$ *HIF-2α*$^{flox/flox}$ mice.

Figure-S3 (Takeda)

a

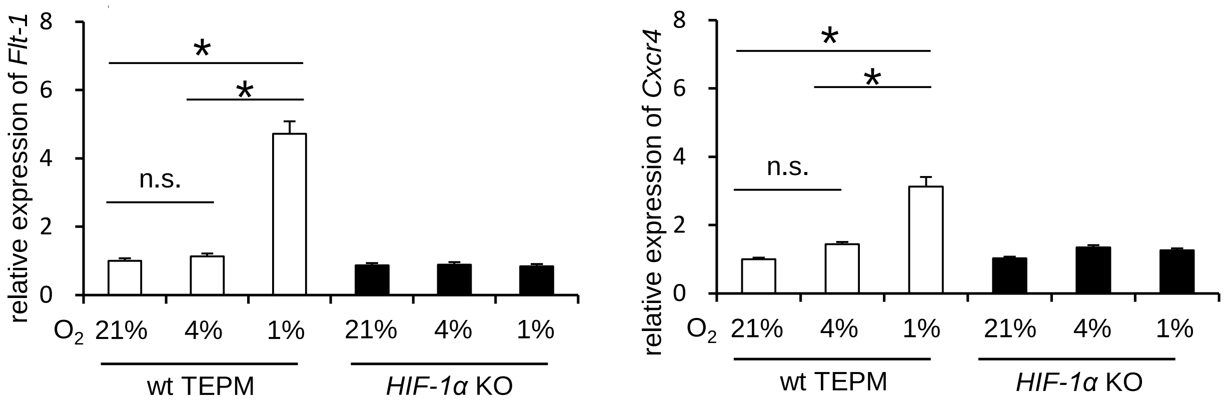

b

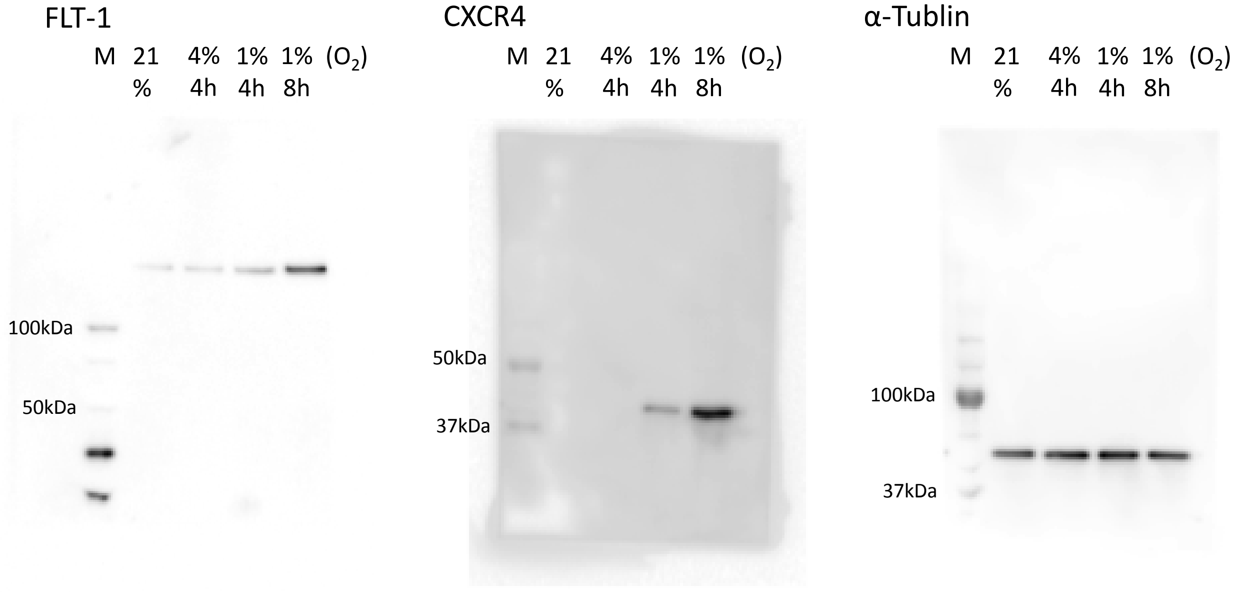

(a) The gene expressions of *Flt-1* and *Cxcr4* in normoxia (21 % O_2, 4h), mild hypoxia (4 % O_2, 4h) and severe hypoxia (1 % O_2, 4h) were assessed by quantitative RT-PCR. n = 3 animals. All graphs indicate average with SD. *, $P < 0.05$; n.s., not significant; wt TEPM, *Tie2-Cre*$^{-/-}$ *HIF-1α*$^{flox/flox}$ mice; *HIF-1α* KO, *Tie2-Cre*$^{+/-}$ *HIF-1α*$^{flox/flox}$ mice.
(b) Immunoblot analysis of FLT-1 and CXCR4 in wild-type TEPMs under each O_2 concentration (21%, 4% 4h, 1% 4h or 1% 8h). α-Tublin was used as a loading control.

Figure-S4 (Takeda)

a

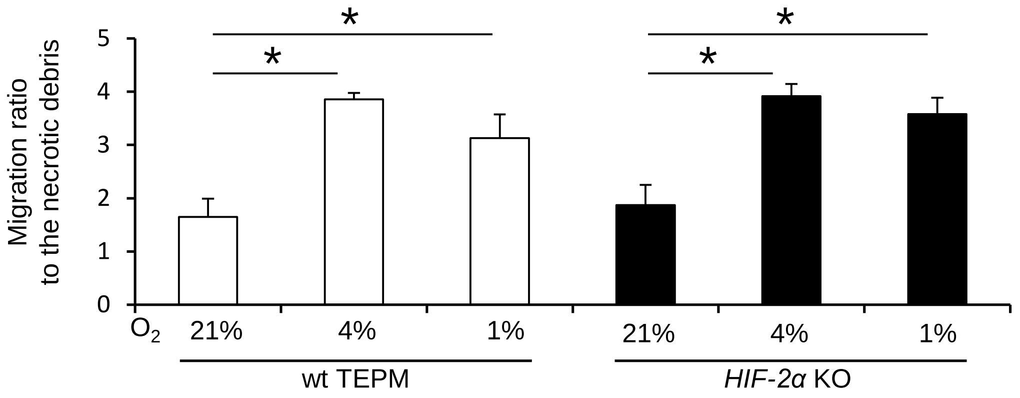

b

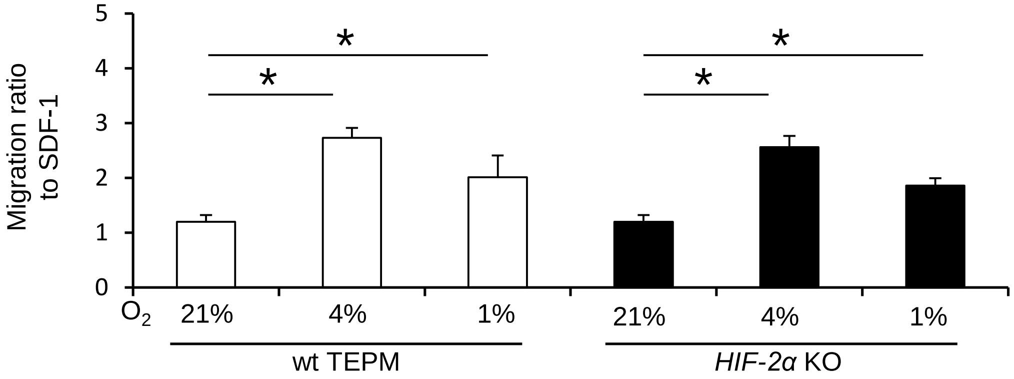

c

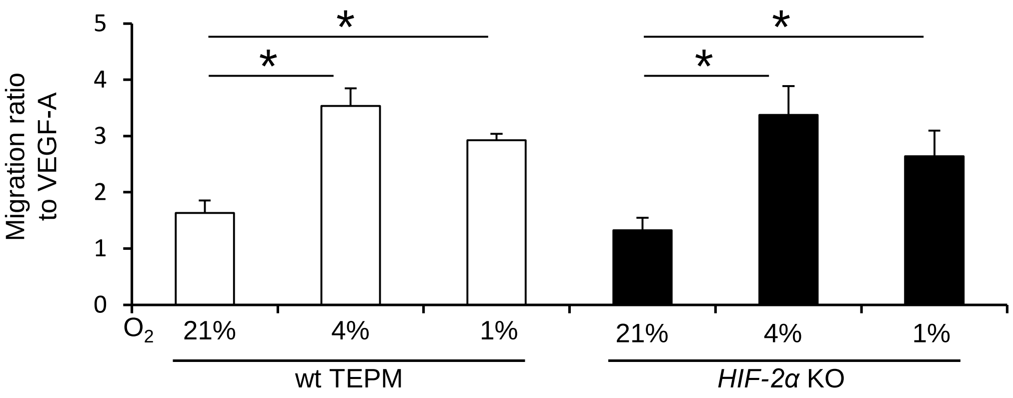

Macrophage motility of control and HIF-1α deficient macrophages to various chemoattractants was measured in the same manner as Figure 4(c). *n* = 3 animals per group. All graphs indicate average with SD. *, $P < 0.05$; wt TEPM, *Tie2-Cre*$^{-/-}$ *HIF-2α*$^{flox/flox}$ mice; *HIF-2α* KO, *Tie2-Cre*$^{+/-}$ *HIF-2α*$^{flox/flox}$ mice.

Figure-S5 (Takeda)

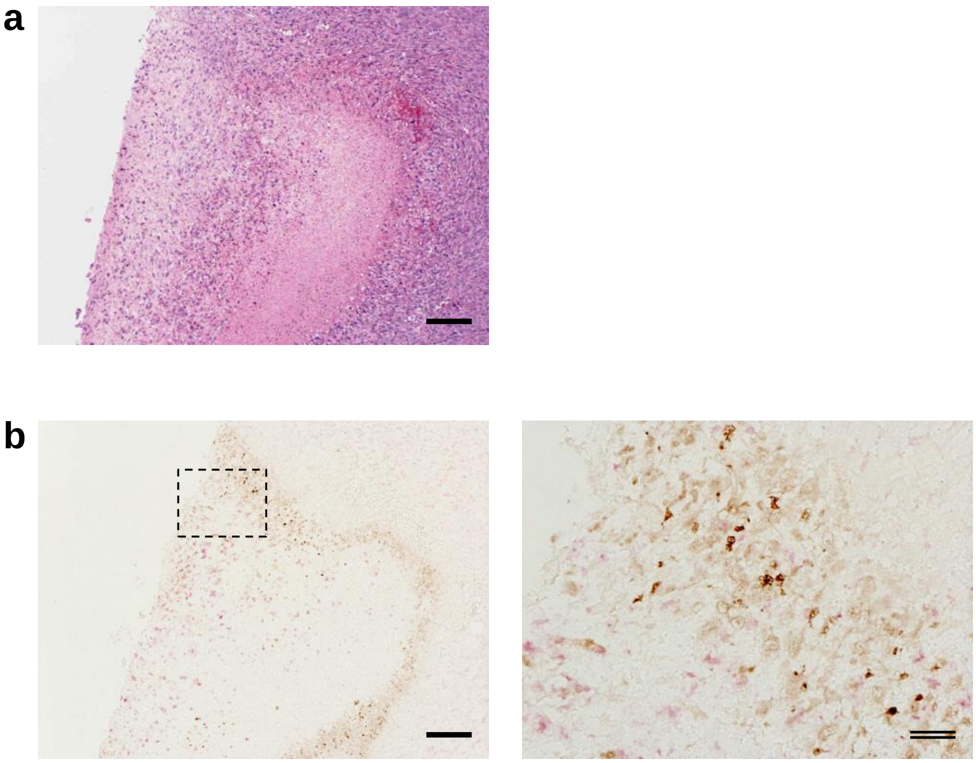

Representative immunohistochemical staining of Hematoxylin and eosin (a), Pimonidazole (brown) and F4/80 (red) (b) in LL2 xenograft model.
Scale bar, single line = 200μm, double line = 50μm.

Figure-S6 (Takeda)

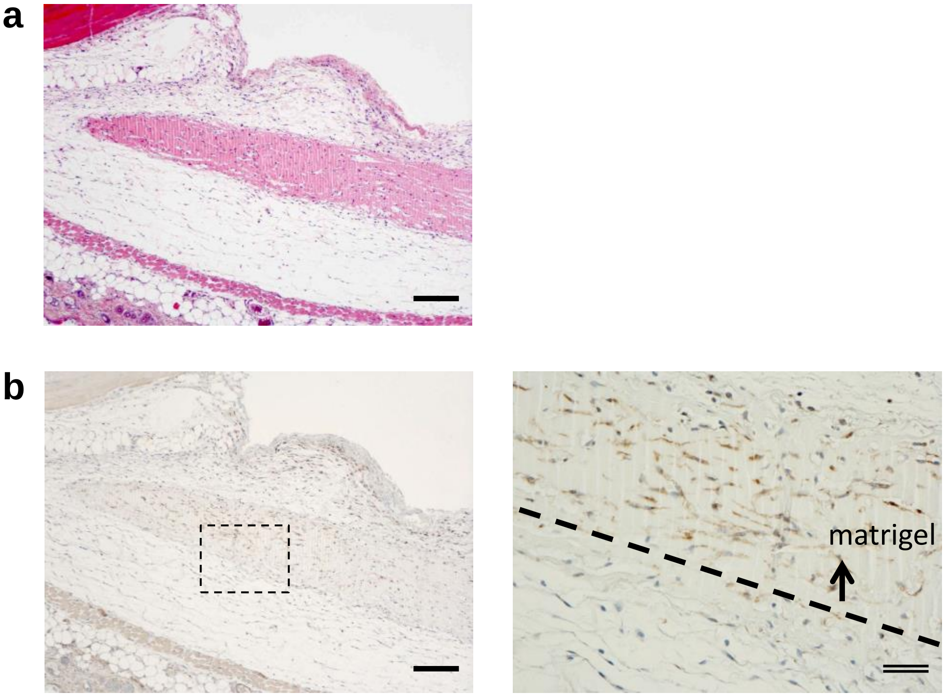

Representative immunohistochemical staining of hematoxylin and eosin (a) and carbonic anhydrase-Ⅸ (CA9) (brown) (b) in *in vivo* migration model. Scale bar, single line = 200μm, double line = 50μm.

Figure-S7 (Takeda)

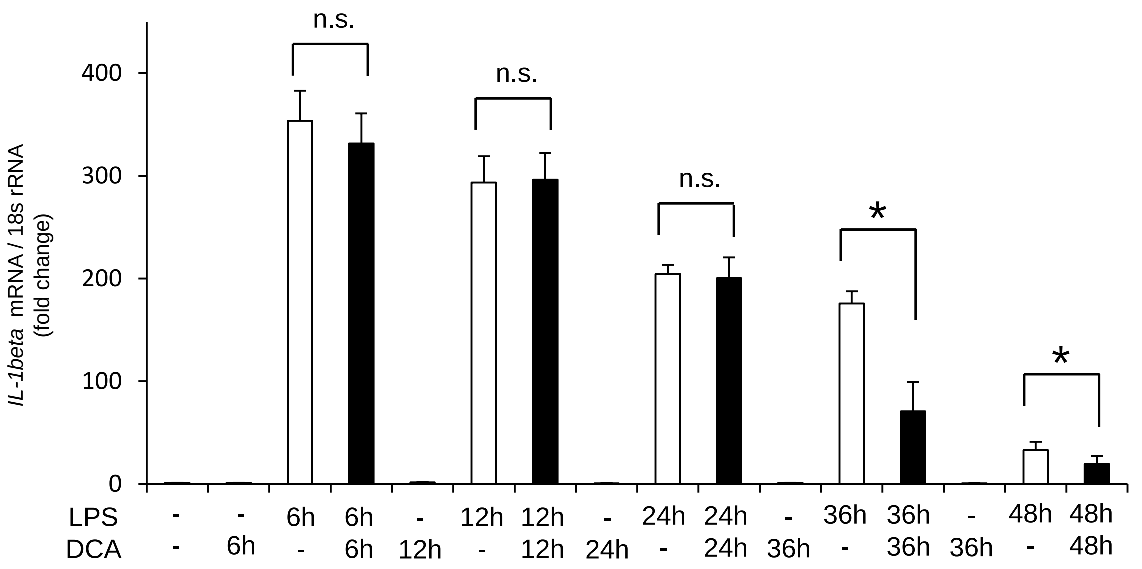

TEPMs were treated with LPS (1 mg/ml) and/or DCA (10 mM) for 6, 12, 24, 36 or 48 hours.
The gene expressions of *IL-1beta was* assessed by quantitative RT-PCR.
The graph indicates average with SD. *, *P* < 0.05; n.s., not significant.
LPS, lipopolysaccharide; DCA, dichloroacetic acid.

Figure-S8 (Takeda)

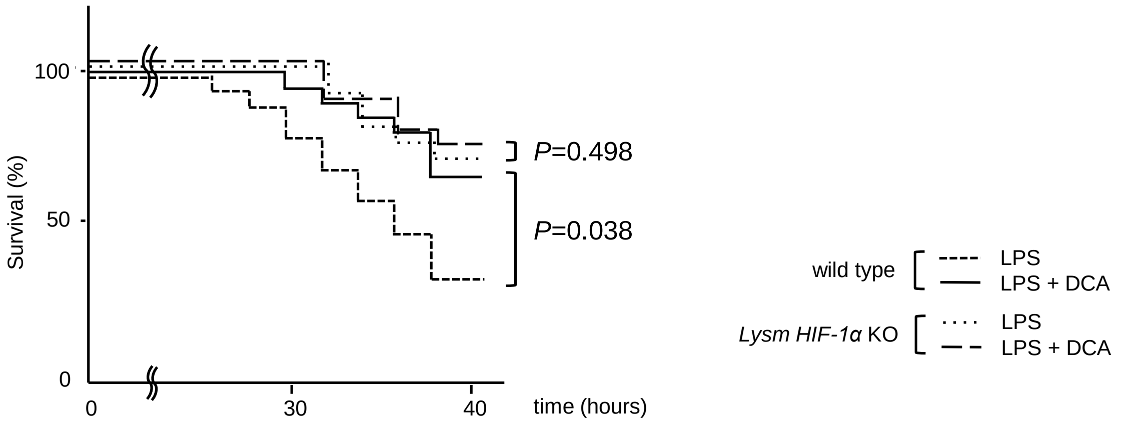

Mice were pre-treated with DCA (100 mg/kg body weight) or PBS. Endotoxin shock was induced 6h after pretreatment by the intraperitoneal injection of LPS (Sigma) (40 mg/kg body weight). *n* = 20 animals per group.
wild type, *Lysm-Cre*$^{-/-}$ *HIF-1*$\alpha^{flox/flox}$ mice; *Lysm HIF-1*α KO, *Lysm-Cre*$^{+/-}$ *HIF-1*$\alpha^{flox/flox}$ mice.

Figure-S9 (Takeda)

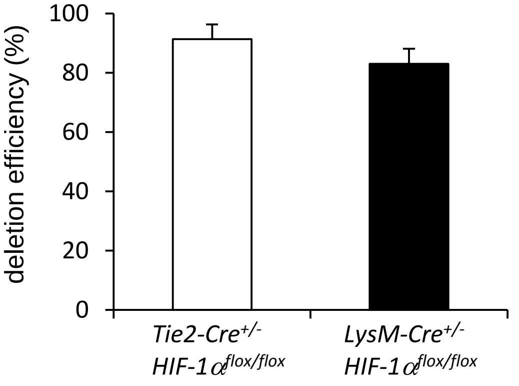

Total RNA was isolated from TEPMs and subjected to real-time PCR with primers spanning the targeted region as well as primers for an undeleted control gene for normalization. Efficiency of deletion was calculated by quantitative PCR. The graph indicates average with SD.

Figure-S10 (Takeda)

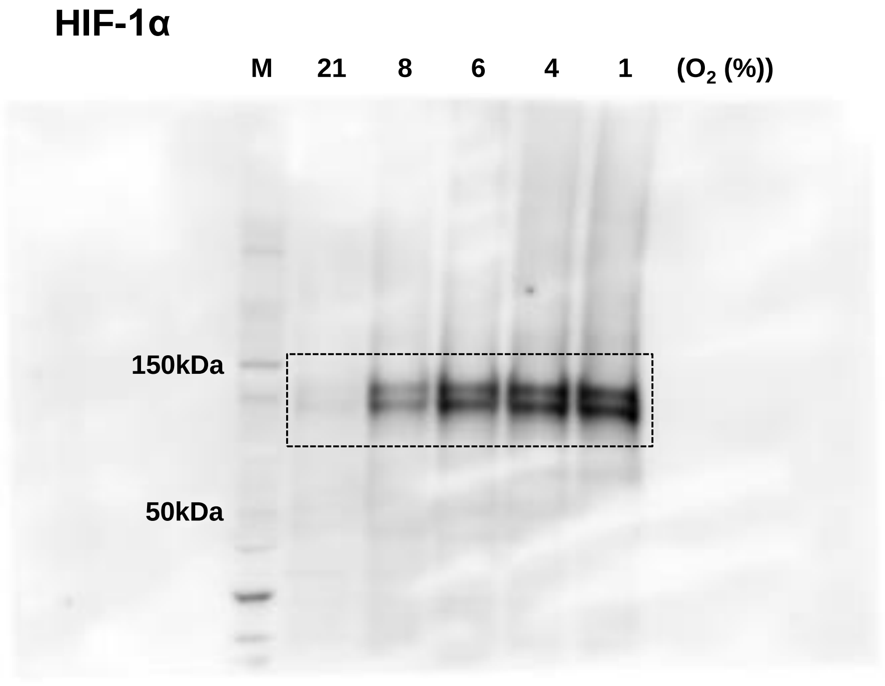

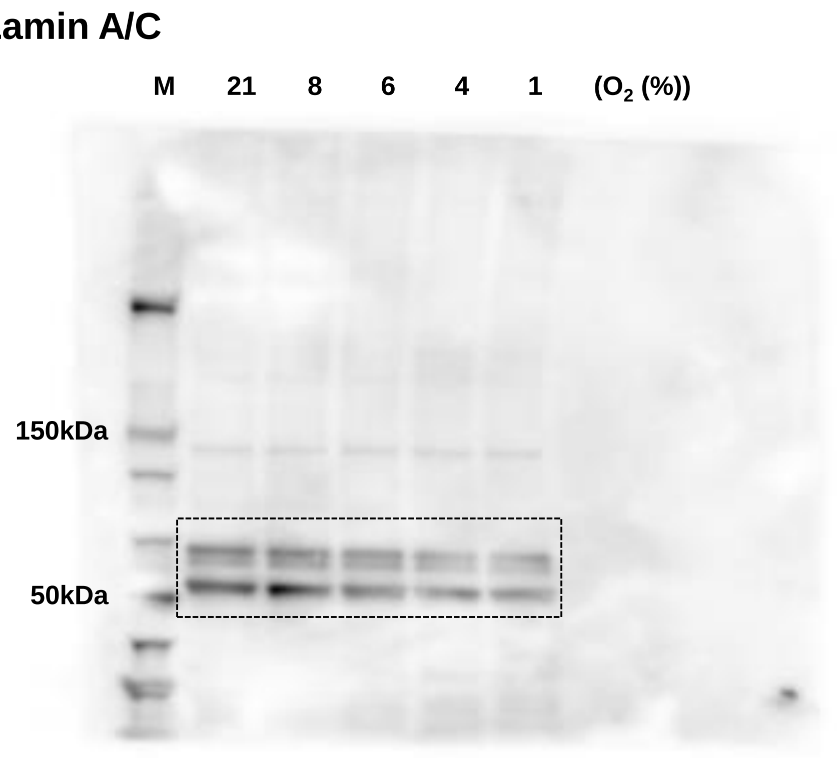

Whole images of western blots.

Figure-S10 (Takeda)

HIF-2α

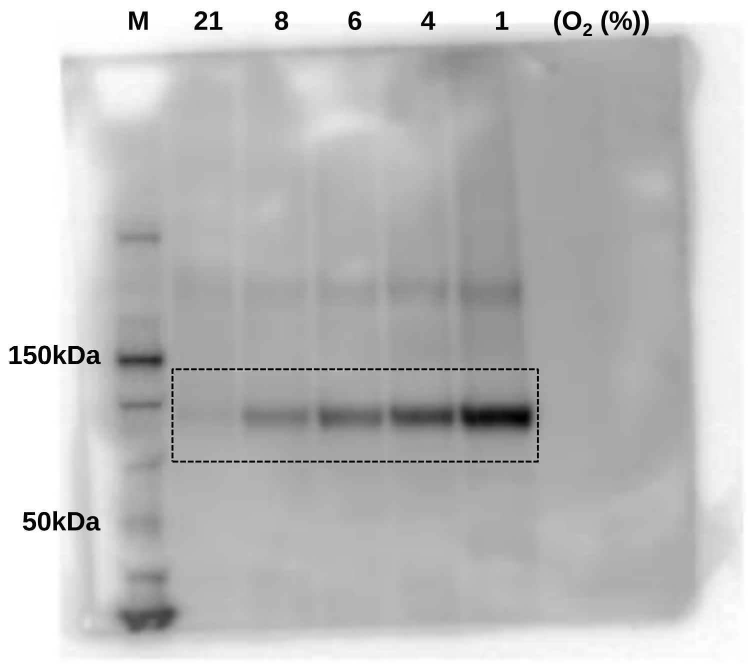

Lamin A/C

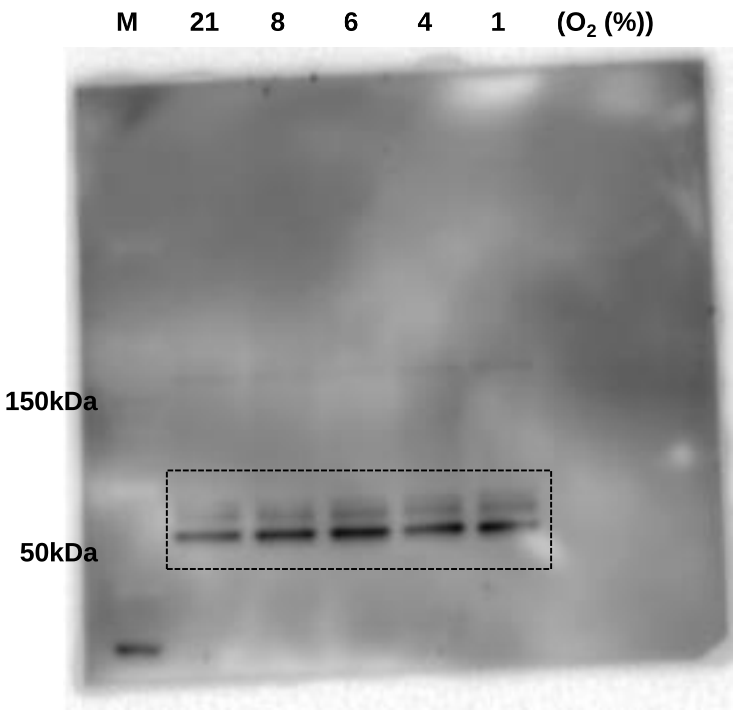

Whole images of western blots.

Supplementary Tables

Table-S1 (Takeda)

The mRNA half-life of hypoxia induced genes

gene	half-life of mRNA (hours) (average ± SD)
Pdk1	3.3 ± 0.5
Eno1	4.3 ± 0.5
Ldha	4.7 ± 0.8
Gapdh	5.3 ± 0.5
Pdk3	5.5 ± 0.4
Pgk1	5.8 ± 0.7
Aldoa	6.3 ± 0.5
Pfk2	6.4 ± 0.4
Tpi1	7.3 ± 0.2
Hk1	7.7 ± 0.5
Pfk1	7.8 ± 0.4
Hk2	8.3 ± 0.5
Glut2	8.5 ± 0.2
Pkm1	9.6 ± 0.4
Glut1	10.1 ± 1.4

Table-S2 (Takeda)

Primer Sequences for RNA analyses

18S	F	CGAAAGCATTTGCCAAGAAT	
18S	R	AGTCGGCATCGTTTATGGTC	
Glut1	F	ATACTCATGACCATCGCGCTAG	
Glut1	R	AAAGAAGGCCACAAAGCCAAAG	
Pgk1	F	CTGTGGTACTGAGAGCAGCAAGA	
Pgk1	R	CAGGACCATTCCAAACAATCTG	
Ldha	F	TGGCAGACTTGGCTGACAG	
Ldha	R	ACCTTCACAACATCCGAGATTC	
Pdk1	F	GCAGCAGAGAGTAAACTGTTTG	
Pdk1	R	TGGTCACCTGACCTCTCG	
Pdk2	F	TGGACCGCTTCTACCTCAG	
Pdk2	R	TCTTTCACCACATCAGACACG	
Pdk3	F	GCCCAAGGCGTGATTGAGTA	
Pdk3	R	GGGTTAGTGTCACCACCAAAC	
Pdk4	F	CTTTAGTTACACGTACTCCACTG	
Pdk4	R	ACAGACTCAGAAGATAAAGCCT	
Nos2	F	ACCCTAAGAGTCACCAAAATGGC	
Nos2	R	TTGATCCTCACATACTGTGGACG	
TNF-α	F	CCATTCCTGAGTTCTGCAAAGG	
TNF-α	R	AGGTAGGAAGGCCTGAGATCTTATC	
IL-1b	F	AAGTGATATTCTCCATGAGCTTTGT	
IL-1b	R	TTCTTCTTTGGGTATTGCTTGG	
Flt-1	F	GGCATCCCTCGGCCAACAATC	
Flt-1	R	AGTTGCTGCTGGGATCCAGG	
CXCR4	F	TGGAACCGATCAGTGTGAGT	
CXCR4	R	GGGCAGGAAGATCCTATTGA	
HIF-1α	F	GAAACGACCACTGCTAAGGCA	
HIF-1α	R	GGCAGACAGCTTAAGGCTCCT	
HIF-1α	F’	GATTCGCCATGGAGGGC	(for checking the deletion efficiency)
HIF-1α	R’	AGACTCTTTGCTTCGCCGAG	(for checking the deletion efficiency)

Primer Sequences for ChIP PCR analyses

Glut*1*	F	ATTTCTAAGGCCCTGGGTCC
Glut1	R	CCTGCCTGATGCGTGTCA
Pgk1	F	CACCTTCTACTCCTCCCCTAGTCA
Pgk1	R	CACGAGACTAGTGAGACGTGCTACTT
Ldha	F	CCATCGTGCACTAGCGGTAC
Ldha	R	TCAGTTCCCCAAAGCACAGTAG
Pdk1	F	ATAGTCGCACGTCCCTGTTAC
Pdk1	R	TGCGTCTTAGGTGCTTCCTTC
Pgk1 distal (negative control)		
	F	GGCATTAGGGCATTCAGTTC
	R	TCCACTCTGAATCCTGGTGA

Primer Sequences to estimate the DNA content

nuclear genome		
Ndufv1	F	CTTCCCCACTGGCCTCAAG
Ndufv1	R	CCAAAACCCAGTGATCCAGC
mitochondria genome		
Cox1	F	TGCTAGCCGCAGGCATTAC
Cox1	R	GGGTGCCCAAAGAATCAGAAC

Table-S3 (Takeda)

Mass accuracy of the used CE–QTOF–MS system for lactate and adenine nucleotide detection. The slope and R2 values of the calibration curves are also shown.

Compound	Formula	*m/z*		Error (ppm)	Calibration curve	
		Theoretical ($[M–H]^-$)	Observed ($[M–H]^-$)		Slope	R2
Lactate	$C_3H_6O_3$	89.0244	89.0249	5.62	715.34	0.99
AMP	$C_{10}H_{14}N_5O_7P$	346.0558	346.0565	2.02	1045.1	0.99
ADP	$C_{10}H_{15}N_5O_{10}P_2$	426.0221	426.0229	1.88	2035.4	0.99
ATP	$C_{10}H_{16}N_5O_{13}P_3$	505.9885	505.9895	1.98	1697.5	0.99
